# Impact of the COVID-19 Pandemic on the Outcomes of a Multifaceted Program on Antibiotic Prescribing in Primary Care Among Children Under Three Years of Age

**DOI:** 10.3390/antibiotics15010101

**Published:** 2026-01-19

**Authors:** Gema Martín-Ayala, Santiago Alfayate-Miguélez, Casimiro Jiménez-Guillén, Manuel Alcaraz-Quiñonero, Antonio Iofrío-De Arce, José Arnau-Sánchez

**Affiliations:** 1 General Directorate of Health Planning, Research, Pharmacy and Citizen Services, Health Counseling of Murcia Region, E-30001 Murcia, Spain; gema.martin@carm.es (G.M.-A.); casimiro.jimenez@carm.es (C.J.-G.); 2 Research Group of Murciano Institute of Biosanitary Research, IMIB, E-30120 Murcia, Spain; 3 National Plan for Antibiotic Resistance (PRAN) in Murcia Region, E-30001 Murcia, Spain; 4 Health Care Center of Yecla, V Health Area, E-30510 Murcia, Spain; 5 Health Care Center “El Ranero”, VI Health Area, E-30009 Murcia, Spain; antonio.iofrio2@carm.es; 6 Nursing Faculty, Health Sciences Campus, University of Murcia, E-30120 Murcia, Spain

**Keywords:** rational antibiotic use, primary care, COVID-19, early childhood, paediatric population

## Abstract

**Background/objective**: Inappropriate antibiotic use in paediatric populations is a leading driver of antimicrobial resistance. In the Murcia Region, Spain, the Purapi program promotes the rational use of antibiotics among children under 3 years of age. This study aimed to analyse antibiotic use in this age group during the pandemic period (2020–2023) and to assess the impact of the COVID-19 pandemic on the effectiveness of a multifaceted program promoting appropriate antibiotic use. **Methods**: A retrospective, multicentre, population-based study was conducted in primary care using data from 2019 to 2024. Systemic antibiotic use (ATC J01 group) among children under three years was measured as defined daily doses per 1000 inhabitants per day (DHD). Differences across years and healthcare areas were assessed using analysis of variance (ANOVA) with Bonferroni correction. **Results**: Antibiotic consumption decreased by 49% in 2020 compared to 2019, coinciding with the implementation of national COVID-19 containment measures. From 2021 onward, a gradual increase was observed; however, by 2024, levels remained 9% below pre-pandemic values. Penicillins account for 75% of prescriptions, mainly amoxicillin and amoxicillin–clavulanic acid. While variability across healthcare areas decreased during the pandemic, variability among primary care centres increased. **Conclusions**: The pandemic resulted in a temporary reduction in antibiotic use, followed by a partial rebound. Ongoing educational and stewardship interventions within the Purapi framework were instrumental in maintaining rational prescribing and may have contributed to maintaining reduced antibiotic consumption among children under three years of age during and after the pandemic. Strengthening and harmonising these initiatives is essential to ensure consistent paediatric antibiotic stewardship in primary care.

## 1. Introduction

Antibiotics are the most commonly prescribed therapeutic agents in general paediatric care, particularly primary care [1]. Most infectious processes in children under three are viral and self-limiting and therefore do not typically require antibiotics, especially upper respiratory tract infections (URTIs) [2,3]. Despite this, families often pressure clinicians to prescribe antibiotics in early childhood [3,4,5]. Excessive and inappropriate use of antibiotics contributes significantly to antibiotic resistance [4] and has also been linked to autoimmune diseases [6] and obesity [7,8].

According to the National Antibiotic Resistance Plan (PRAN), high rates of antibiotic consumption among children under five are a significant concern in Spain. These rates are higher than those in other European countries for the same age group. Prescribing antibiotics when there is no bacterial cause is common, for example, in cases of viral pharyngotonsillitis, bronchitis, and the common cold. In addition to unnecessary prescriptions, antibiotic selection is also frequent. There is a high use of amoxicillin–clavulanic acid and macrolides, even when they are not the first-line choices [9].

The COVID-19 pandemic significantly affected public health, especially among children. Exceptional circumstances, such as lockdowns, widespread mask use, limited healthcare access, and the school and nursery closures, altered disease transmission patterns and disrupted the delivery of healthcare services. In the Region of Murcia, schools moved to online instruction on 16 March 2020, and reopened in September. These changes led to an increase in telephone consultations and a reduction in face-to-face visits, making diagnoses during this period less reliable [10,11,12,13].

In Spain, SARS-CoV-2 began circulating in February 2020 and was declared a notifiable disease during the state of emergency (Royal Decree 463/14 March 2020). Incidence rates remained relatively stable throughout 2020, with slight increases in late autumn. In 2021, modest rises were observed, followed by another increase from July to September. In 2022, the incidence rates peaked from January to March, then declined through August and stabilised at low levels. The usual seasonal patterns of influenza and Respiratory Syncytial Virus (RSV) circulation were disrupted, leading to atypical off-season peaks. From 2023 onward, these patterns gradually returned to normal [14].

In response to the pandemic, Spain implemented extensive public health measures. These included nationwide lockdowns, mandatory mask use, and the closure of educational institutions. Additional restrictions included limited access to hospitals and primary care, curfews, and caps on gatherings and venue capacity. The intensity of these measures varied over time in response to epidemiological indicators, including infection rates, mortality, vaccination coverage, and population immunity levels. The state of emergency concluded in July 2023, when mask mandates in healthcare settings were lifted [15].

In the Region of Murcia, a regional primary care program began in 2015 to optimise antibiotic use for upper respiratory tract infections (URTIs) in children under three years of age (Purapi Program). This ongoing initiative has implemented educational interventions directed at paediatric prescribers, family physicians, emergency physicians (SUAPs), nurses, pharmacists, and the general population. As a result, antibiotic consumption was reduced by 48% between 2015 and 2019 [16].

The Purapi program was developed using a structured, mixed-methods methodological framework. A qualitative component, grounded in Glaser and Strauss’ theory-based approach, involved focus group discussions with 25 paediatricians from the nine healthcare areas to identify barriers, facilitators, behavioural drivers, and contextual factors influencing prescribing practices. These insights served as the basis for a quantitative, quasi-experimental, non-randomised longitudinal design assessing antibiotic consumption in children under three years of age using DHD indicators. The program was implemented sequentially in four phases: (1) situational analysis, (2) design of targeted interventions, (3) a pilot phase in three primary care centres with diverse baseline consumption profiles, and (4) scaling up to all 87 primary healthcare centres in the Region of Murcia.

This study was designed to analyse antibiotic use during the pandemic period (2020–2023) in children under three years of age and to assess how the COVID-19 pandemic influenced the outcomes of a multifaceted program promoting the rational use of antibiotics.

## 2. Results

**Figure 1:**
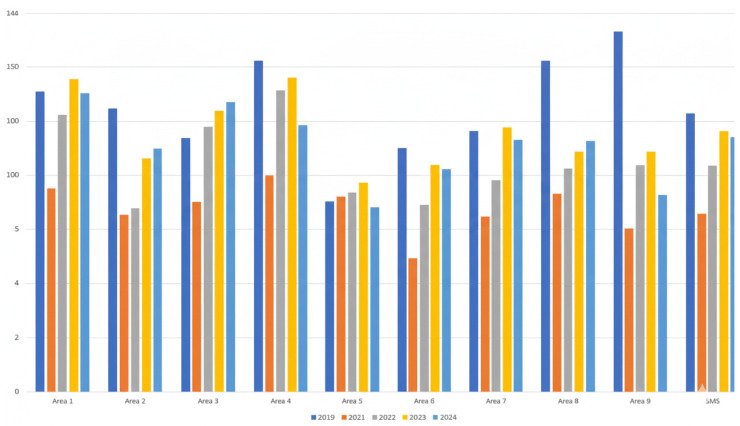
Changes in the consumption of systemic antibiotics (J01). DHD: defined daily doses per 1000 inhabitants per day across all healthcare areas (HCAs) of the Murcia Region. Abbreviations: DHD, defined daily doses per 1000 inhabitants per day; HCAs, healthcare areas; SMS, Murciano Health Service.

The Region of Murcia is administratively divided into nine healthcare areas (HCAs): Murcia Oeste, Murcia Este, Cartagena, Lorca, Altiplano, Vega Media del Segura, Noroeste, Mar Menor, and Vega Alta. These areas include both urban and rural settings and together represent the full regional coverage of primary care services. All nine HCAs were included in the analysis. Across all HCAs, antibiotic use (J01) decreased by 49% in 2020 compared to 2019. From 2021 to 2023, consumption increased modestly, resulting in an 85% rise relative to 2020 levels. When comparing pre- and post-pandemic data (2019 vs. 2024), an overall decrease of 9% was observed; however, this reduction was not statistically significant (*p* = 0.55). The data indicated a downward trend in antibiotic use (Table 1, Figure 1). This pattern remained consistent throughout the study period.

To further investigate consumption trends, an analysis of variance (ANOVA) was conducted to compare mean antibiotic consumption (DHD) across study years (2019–2024) and HCAs. Statistically significant differences were observed both across years and among areas (*p* < 0.001). Post hoc pairwise comparisons using 2019 as the reference year revealed significant reductions in 2020 and 2021, whereas differences for 2022–2024 were not statistically significant (Table 2).

**Figure 2:**
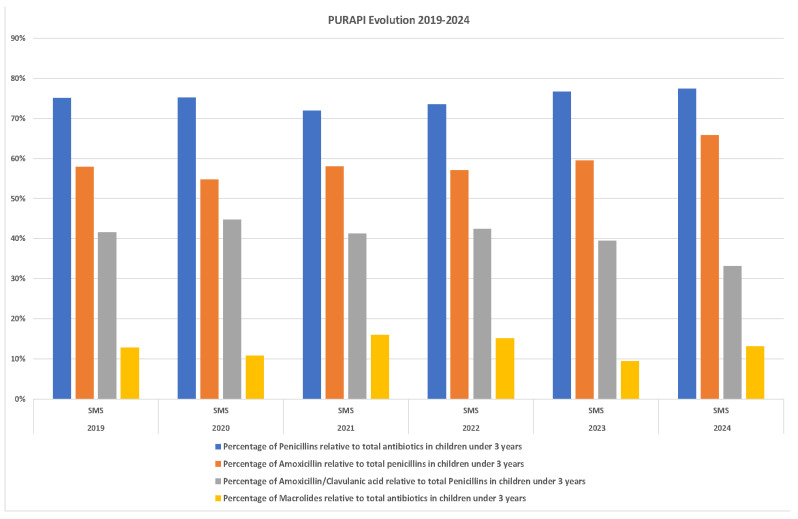
Distribution of the most frequently prescribed antibiotics for common illnesses in infants under 3 years of age. Abbreviations: PURAPI: Program for the Responsible Use of Antibiotics in Early Childhood; SMS: Murciano Health Service.

Penicillins (J01C) were the most commonly prescribed class of antibiotics, accounting for approximately 75% of all prescriptions. Amoxicillin (J01CA04) and amoxicillin–clavulanic acid (J01CR02) were the predominant agents throughout the study period. Although the use of penicillins declined slightly in 2021, it subsequently surpassed pre-pandemic levels. Amoxicillin use decreased in 2020 compared to 2019 but subsequently rebounded and exceeded previous levels, whereas amoxicillin–clavulanic acid showed a continuous decline throughout the study period (Table 3; year-by-year percentages not shown, Figure 2).

Compared to 2019, macrolide use increased by approximately 40% from 2020 to 2022 (n, decreased by 37% from 2022 to 2023, and rose again in 2024 to levels exceeding those observed before the pandemic (Table 3, Figure 2).

**Figure 3:**
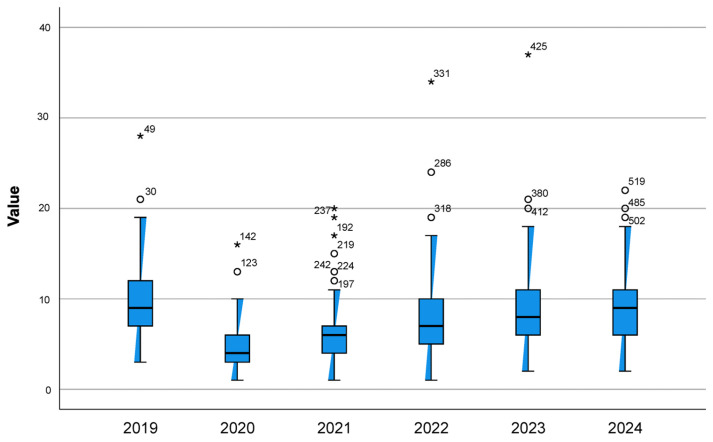
Minimum and maximum antibiotic consumption (DHD) across healthcare areas (HCAs) in children under 3 years of age, 2019–2024. Numbers indicate anonymised identifiers of units presenting extreme values. Asterisks indicate outlier values. Circles represent minimum and maximum values. Abbreviations: DHD, defined daily doses per 1000 inhabitants per day; HCAs, healthcare areas.

**Figure 4:**
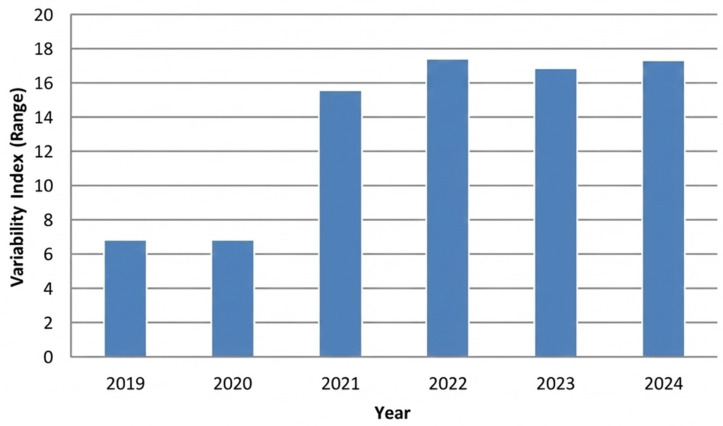
Range (minimum and maximum) of antibiotic consumption (DHD) across primary care centres for children under 3 years of age, 2019–2024. Abbreviations: DHD, defined daily doses per 1000 inhabitants per day.

Regarding variability, Figure 3 illustrates the minimum and maximum antibiotic consumption (DHD) across HCAs for children under three years of age during the study period (2019–2024). Mean consumption decreased from 1.9 in 2019 to 1.5 in 2024, with a marked decline in 2020 and 2021, followed by a progressive recovery to pre-pandemic levels by 2024. In contrast, variability among individual primary care centres within each area was greater (ranging from 6.8 to 17.4), remained stable during the pre-pandemic years, and reached its lowest point at the onset of the pandemic (6.8). From 2021 onwards, variability increased substantially, peaking in 2022 (17.4), and persisted until the end of the study period (Figure 4).

## 3. Discussion

The present study aimed to analyse antibiotic consumption among children under three years of age in the Region of Murcia during the COVID-19 pandemic and to assess the impact of the pandemic on the outcomes of a multifaceted program promoting the rational use of antibiotics in primary care. Overall, our findings showed a marked reduction in antibiotic use during the first year of the pandemic, followed by a partial recovery in subsequent years. However, consumption levels remained below pre-pandemic levels by 2024. Penicillins, particularly amoxicillin and amoxicillin–clavulanic acid, continued to be the most frequently prescribed antibiotics, while prescribing variability across healthcare areas decreased during the pandemic.

A comparison of antibiotic consumption between the pre-pandemic year (2019) and the first year of the pandemic (2020) revealed a 49% reduction. This decrease was similar to reductions reported in other Spanish regions [17]. International studies have documented even greater declines, reporting reductions of 53% [18], 80% [19], and up to 90% [20]. In the present study, this marked reduction may be attributed to a substantial decrease in URTIs among children, which coincided with public health measures implemented by the Spanish Government during the state of alarm declared in March 2020 in response to the SARS-CoV-2 pandemic. These findings are consistent with previous reports describing reduced circulation of respiratory pathogens during this period [17,21,22,23]. Additional contributing factors may include the expansion of telemedicine and parental reluctance to attend healthcare facilities due to fear of infection.

During the pandemic period (2020–2023), antibiotic consumption increased progressively but remained below pre-pandemic levels (2019). This upward trend has also been reported internationally [11,24,25]. Several factors may have contributed to this gradual increase, including the gradual relaxation of public health restrictions, reduced adherence to social distancing measures, the re-emergence of seasonal respiratory viruses following epidemiological shifts during the pandemic, and the continued use of telephone consultations as a routine healthcare delivery method [26,27,28]. Notably, a Danish study found that telephone consultations were associated with significantly higher rates of antibiotic prescribing compared to face-to-face visits [29].

Throughout the pandemic, the Purapi program maintained interventions targeting healthcare professionals and the general population through both in-person and virtual formats. These included online training seminars on responsible antibiotic use for prescribers, dissemination of educational materials via the regional healthcare digital platform (https://www.escueladesaludmurcia.es/escuelasalud/cuidarse/pediatria/guiasanticipatorias.jsf, accessed 20 October 2025), and online educational workshops for families on managing common paediatric infectious diseases. The sustained implementation of these initiatives likely helped preserve the reductions in antibiotic use achieved during the pre-pandemic period (2015–2019) [16].

A comparison between pre-pandemic and post-pandemic years revealed a reduction in mean antibiotic consumption from 10.33 DHD to 9.42 DHD, representing an approximate 9% decrease that remained below 2019 levels. However, this difference was not statistically significant and should therefore be interpreted with caution. These trends differ from those reported in several European countries, where antibiotic consumption increased after the pandemic compared to pre-pandemic values [26,30,31]. Although causal inference cannot be established, the continued implementation of antimicrobial stewardship and educational interventions within the Purapi program may have helped prevent a more pronounced post-pandemic rebound and contributed to maintaining relatively stable consumption levels.

Amoxicillin (J01CA04) was the most frequently used antibiotic. Its use declined modestly in the first pandemic year compared to the previous year (−5.5%), then returned to pre-pandemic levels and exceeded them in later years. Conversely, amoxicillin–clavulanic acid (J01CR02) use increased slightly during the first year of the pandemic; however, in subsequent years, it showed a downward trend, resulting in a 12% reduction over the course of the pandemic. Overall, the reduction reached 20% between 2019 and 2024. These findings are consistent with those reported in other studies [17,19]. Given that most primary care infections are caused by microorganisms that do not produce β-lactamases, amoxicillin–clavulanic acid should be reserved for cases where the likely etiological agent is a β-lactamase producer, particularly given its association with adverse effects, such as *Clostridioides difficile* infection and acute hepatotoxicity.

Compared with studies reporting increased use of azithromycin and other macrolides during the pandemic—often exceeding pre-pandemic levels [32]—our study found an initial reduction in macrolide consumption in 2020 compared to 2019 (12.73% vs. 10.79%). This was followed by a variable pattern in subsequent years, with a temporary decrease in 2023 and a renewed increase in 2024 This pattern may be partly explained by the relaxation of public health measures from 2022 onwards and the resurgence of viral infections such as RSV, influenza, and rhinovirus, which may have altered the circulation dynamics of respiratory pathogens In addition, increased macrolide use during this period has been linked to therapeutic hypotheses suggesting a potential role of azithromycin in SARS-CoV-2 infection, as reported elsewhere [26,32,33]. Within this context, training strategies implemented within the Purapi framework, aimed at improving prescribing practices among healthcare professionals and aligned with the National Health Systems’ Antimicrobial Therapeutic Guide [34], appear to have been effective in promoting more appropriate antibiotic use during the early stages of the pandemic.

Previous research in this setting identified significant variability in antibiotic consumption across HCAs and primary care centres [16]. During the period analysed, variability among HCAs decreased during the first two years of the pandemic compared to 2019 (1.9 vs. 1.5). In contrast, variability among primary care centres increased substantially, beyond pre-pandemic levels (6.8 vs. 17.3). To our knowledge, few studies have investigated prescribing variability in children under three years of age. Several hypotheses may explain these findings. The relative homogeneity in antibiotic consumption across HCAs may be related to the reduced incidence of respiratory infections following public health measures during the pandemic, such as lockdown, mask use and improved hygiene, which decreased overall diagnostic pressure. Conversely, the greater heterogeneity observed among primary care centres may reflect contextual and professional differences. The expansion of telephone consultations and reduced access to diagnostic support during the pandemic may have amplified individual clinical decision-making under conditions of uncertainty. In this context, greater clinical experience has been associated with more conservative approaches to prescribing, whereas less experienced clinicians may be more inclined to use antibiotics empirically “just in case” [35].

In addition, organisational and contextual factors, such as differences in workload, staffing stability, and the rural or urban setting of healthcare centres, may have contributed to the observed variability [30,35,36]. The research team is currently working to identify the specific factors influencing this variability within the region.

This study has several limitations. First, prescriptions issued through private healthcare providers or mutual insurance entities were not included; however, these represent only a minority in our setting. Second, although aggregate data on antibiotic consumption within the J01 group were analysed, it was not possible to link specific antibiotics prescribed to their corresponding diagnoses due to limitations in the coding processes of the Murciano Health Service information system. The research team acknowledges that establishing this link would provide valuable insights into the quality of prescribing practices. Nevertheless, previous studies conducted in Spain have shown that URTIs are the most common reason for antibiotic prescribing in children under three years of age [17]. In addition, due to the observational nature of this study and the absence of a control group, causal inferences regarding the direct impact of the Purapi program on antibiotic prescribing cannot be established. Finally, analysis of variance was appropriate for comparing mean annual antibiotic consumption across years and healthcare areas. More complex time-series approaches could be explored in future studies, particularly with higher temporal resolution data.

Despite these limitations, the broad coverage of this study makes its findings generalisable to other regions with similar characteristics. Furthermore, the ongoing involvement and support of all stakeholders, including paediatricians, family physicians, pharmacists, the general population, and the regional health administration, should be highlighted. These factors are highly favourable for ensuring the sustainability of the results over time.

## 4. Materials and Methods

### 4.1. Study Design

A retrospective, multicentre, observational study was conducted involving the paediatric population attending primary healthcare services in the Region of Murcia, Spain.

### 4.2. Study Setting

The Region of Murcia, located in southeastern Spain, is one of the country’s 17 autonomous communities and has a population of approximately 1.5 million inhabitants. Primary care services are organised into nine healthcare areas comprising 87 basic health zones, with paediatric care provided by 252 primary care paediatricians. As of 1 January 2019, Murcia had a population of 1,467,281 inhabitants (including 45,415 children under 3 years of age), which increased to 1,568,492 inhabitants in subsequent years [37].

### 4.3. Study Population

The study population included all children under three years of age in the Region of Murcia who were prescribed antibiotics for outpatient management of upper respiratory tract infections (URTIs), such as the common cold, sore throat, pharyngitis, and acute otitis media (AOM), during the study period.

### 4.4. Quantitative Assessment of Antibiotic Consumption

This study sought to quantify yearly outpatient use of systemic antibacterial agents belonging to group J01 of the Anatomical Therapeutic Chemical (ATC) classification among children aged 0–3 years in the Region of Murcia over the period 2019–2024. Analyses were restricted to systemic antibacterials, excluding non-systemic antibiotics and other systemic anti-infective drug classes, including antifungals (J02), antimycobacterials (J04), and antivirals (J05).

### 4.5. Information Consumption Data Collection

In the Region of Murcia, systemic antibacterial agents prescribed for outpatient care can only be obtained with a medical prescription. All prescriptions are generated electronically and systematically recorded in the regional and national Prescription Centre, which operates as a centralised database within the FACETA system (Pharmacy Information System of the Department of Health), an internal institutional information system of the Murcian Health Service.

Data on antibiotic consumption were sourced from the Pharmaceutical Management Service of the General Directorate of Hospital Care of the Murcian Health Service. Information was extracted from the monthly billing records of official medical prescriptions and included the number of packages (vials) of systemic antibacterial agents dispensed by community pharmacies in the Region of Murcia and reimbursed by the regional health system between 2019 and 2024 based on prescriptions for patients aged 0 to 3 years.

We described the annual rates of antibiotic prescriptions and identified the most consumed antibiotics per 1000 individuals during the study period (DHD).

### 4.6. Measurement and Indicators of Consumption

Antibiotic use was quantified using the defined daily dose (DDD), the standard technical unit recommended by the World Health Organisation for drug utilisation studies. The DDD corresponds to the assumed average maintenance dose per day for a drug when used for its principal indication in adults. For analytical purposes, consumption was expressed as defined daily doses per 1000 inhabitants per day (DHD), calculated as DHD = (number of DDDs × 1000)/(population × 365). This metric allows for standardised comparisons over time and inherently adjusts for annual variations in the population of children under three years of age.

### 4.7. Statistical Analysis

As antibiotic consumption followed a normal distribution and was treated as a continuous variable, descriptive statistics were summarised using means and standard deviations (SD) or 95% confidence intervals (CI). Differences in antibiotic consumption across the study period (2020–2024) were evaluated using analysis of variance (ANOVA), with HCAs and DHD years as between-subjects and within-subjects factors. When ANOVA detected overall differences, post hoc comparisons with Bonferroni correction were performed using 2019 as the reference year. Statistical significance was set at *p* < 0.05. Data analysis was conducted using IBM SPSS Statistics for Windows, version 21.0 (IBM Corp., Armonk, NY, USA).

### 4.8. Ethical Consideration

Although the study was based on secondary registry data and Ethics Board approval was not required under Spanish legislation, the study was approved by the Clinical Research Ethics Committee of the Region of Murcia (code 2017-06-PI). Informed consent was waived due to the population-based nature of the study.

### 4.9. Use of Generative Artificial Intelligence

Generative artificial intelligence tools were used exclusively for language editing purposes, including grammar, clarity, and stylistic improvements. These tools were not used for data analysis, data interpretation, or the generation of scientific content. All AI-generated outputs were reviewed and approved by the authors.

## 5. Conclusions

The evolution of antibiotic consumption among children under three years of age during the pandemic period reflects trends observed in most Spanish regions and across Europe. There was a marked decline in 2020–2021, followed by a subsequent increase in the following years. While public health measures played a significant role in reducing antibiotic use, educational initiatives and antimicrobial stewardship strategies within the Purapi program appear to have been effective in sustaining rational prescribing practices. They may have contributed to limiting a more pronounced rebound. Nonetheless, these findings highlight the need to further strengthen and harmonise interventions to achieve greater uniformity in prescribing practices across the Murcia Region.

## Figures and Tables

**Table 1:** Mean (±SD) outpatient antibiotic consumption (DHD) in children under 3 years of age across nine healthcare areas (HCAs) of the Murcia Region, 2019–2024.

Variable	Years	Total HCAs (*n* = 9)	Mean ± SD	Across Years	Across Areas
DHD	2019		10.33 ± 4.00		
DHD	2020		5.29 ± 2.38		
DHD	2021		6.72 ± 3.56		
DHD	2022		8.50 ± 4.63		
DHD	2023		9.79 ± 4.78		
DHD	2024		9.42 ± 3.75	<0.001	<0.001

Notes. Values are presented as mean ± standard deviation (SD). *p*-values correspond to global comparisons performed using analysis of variance (ANOVA). Significant differences were observed both across study years (2019–2024) and across healthcare areas, with *p* < 0.001 for both comparisons. “Across years” refers to differences in mean antibiotic consumption across the study years (2019–2024). “Across healthcare areas” refers to differences across the nine healthcare areas of the Murcia Region. Bonferroni’s correction was applied for multiple comparisons. Abbreviations: SD, Standard Deviation; DHD, defined daily doses per 1000 inhabitants per day; HCAs, healthcare areas.

**Table 2:** Mean and Standard Deviation (SD) of consumption of antibiotics in children under 3 years of age across the study period.

Variable	2019-Year	Total HCAs (*n* = 9)	Mean Differences	*p* Value	95% Confidence Intervale
DHD	2020		5.03	<0.001	3.39–6.67
DHD	2021		3.6	<0.001	1.96–5.23
DHD	2022		1.82	0.19	0.18–3.46
DHD	2023		0.53	0.940	−1.1–2.1
DHD	2024		0.90	0.55	−0.73–2.5

Notes: Mean differences represent changes in antibiotic consumption relative to the reference year (2019). *p*-values and 95% confidence intervals correspond to post hoc comparisons derived from analysis of variance (ANOVA). Bonferroni correction was applied for multiple comparisons. Abbreviations: SD, Standard Deviation; DHD, defined daily doses per 1000 inhabitants per day; HCAs, healthcare areas.

**Table 3:** Most frequently prescribed antibiotics for common illnesses in infants under 3 years of age.

Year	Penicillins (% Antibiotics)	Amoxicillin (% Penicillins)	Amoxicillin–Clavulanic (% Penicillins)	Macrolides (% Antibiotics)
2019	75.10	57.87	41.58	12.73
2020	75.23	54.70	44.77	10.79
2021	71.91	58.05	41.29	15.90
2022	73.51	57.02	42.38	15.06
2023	76.73	59.54	39.42	9.38
2024	77.46	65.78	33.11	13.04

Notes: Percentages for penicillins and macrolides are calculated as a proportion of total antibiotic prescriptions. Percentages for amoxicillin and amoxicillin–clavulanic acid are calculated as a proportion within the penicillin class. Abbreviations: % antibiotics, proportion of total antibiotic prescriptions; % penicillins, proportion within the penicillin class.

## Data Availability

Data are contained within the article. Additional aggregated data may be available from the corresponding author upon reasonable request, subject to institutional and legal restrictions.

## Abbreviations

The following abbreviations are used in this manuscript:

AB	Antibiotics
ANOVA	Analysis of Variance
AOM	Acute Otitis Media
ATC	Anatomical Therapeutic Chemical Classification System
CI	Confidence Interval
DHD	Defined Daily Doses per 1000 inhabitants per day.
DDD	Defined Daily Doses.
FACETA	Pharmacy Information System of the Department of Health
HCAs	Healthcare Areas
PRAN	National Antibiotic Resistance Plan (Spain)
PURAPI	Program for the Responsible Use of Antibiotics in Early Childhood
RSV	Respiratory Syncytial Virus
SD	Standard Deviation
SMS	Murciano Health Service
SUAPs	Primary Care Emergency Services
URTIs	Upper Respiratory Tract Infections
